# Cross-Species Validation of Pigeon-Specific CHD1 Primers for Molecular Sexing in Pet Birds

**DOI:** 10.3390/ijms262211142

**Published:** 2025-11-18

**Authors:** Simona Marc, Oana Maria Boldura, Cristina Paul, Maria Roberta Tripon, Gabriel Otavă, Jelena Savici

**Affiliations:** 1 Faculty of Veterinary Medicine, University of Life Sciences “King Mihai I” from Timișoara, Calea Aradului 119, 300645 Timișoara, Romania; simona.marc@usvt.ro (S.M.); gabrielotava@usvt.ro (G.O.); jelenasavici@usvt.ro (J.S.); 2 Faculty of Chemical Engineering, Biotechnologies and Environmental Protection, Politehnica University Timișoara, Vasile Pârvan 6, 300223 Timișoara, Romania; 3 Faculty of Engineering and Applied Technologies, University of Life Sciences “King Mihai I” from Timișoara, Calea Aradului 119, 300645 Timișoara, Romania; roberta.tripon@usvt.ro

**Keywords:** molecular sexing, cross-species test, pet birds, PCR

## Abstract

Young and adult birds of a large number of species are sexually monomorphic. The development of molecular methodologies for sexing birds has overcome these difficulties, allowing reliable sex differentiation. An important gene in sex determination across a variety of bird species is the *CHD1* gene, which encodes Chromodomain-helicase DNA binding protein-1 and is located on the avian Z and W chromosomes. The aim of the study is to evaluate the cross-species performance of pigeon-specific CHD1 primers in identifying the molecular sex of birds from five different families, alongside the universal CHD1F/CHD1R primers. The samples were collected from birds of different ages from five different families (*Psittaculidae*, *Psittacidae*, *Columbidae*, *Fringillidae*, and *Phasianidae*). Using universal primer sets, the PCR products that were electrophoresed in agarose gel revealed an average size of 510 pb for the *CHD1* gene on the Z chromosome, while females had two bands with one of 330 pb for the *CHD1* gene on the W chromosome. When pigeon primers were used, the PCR products showed a single band of an size average of 470 pb for males, and two bands in females, with one of them measuring 320 pb. Even though there were small variations in fragment sizes resulting from species-specific intronic differences, these preliminary findings suggest that pigeon CHD1 primers can be used for sexing birds of professional interest with minimally invasive sample collection.

## 1. Introduction

For all species, knowing sex is part of the basic information. In birds, this aspect is not easy to achieve in all situations. There are about 10,400 avian species [1] and approximately 60% of them, at maturity, do not show obvious sexual dimorphism and/or have a monochromatic appearance [2,3,4]. The identification of sex in birds is essential in situations such as managing and conserving avian wildlife, population behavioural studies, ecological studies, and improving captive breeding programmes, among others [2,3,4].

In recent years, the importance of accurate sex determination has extended beyond wildlife studies as the popularity of birds as pets has increased. The pandemic has probably caused a rise in the desire to have birds as pets in recent years. According to the American Pet Products Association in 2021–2022, 8% of US households (5.7 million homes) have pet birds, and about 50% of owners said the pandemic was a factor in getting a feathered companion [5]. In Europe, Italy ranks first, with approximately 12.88 million pet birds in 2023, followed by France with approximately 5.8 million, while Romania has 296,000 pet birds [6].

There are many methods by which the sex of birds can be determined, such as behavioural study, differences in morphological traits (e.g., in poultry the differences in the growth of primary and secondary remiges or flight feathers, which can be observed in the first days after hatching; both rows of a male’s wings are equal in length, while in the female the upper row does not cover the lower one) [7,8,9], acoustic sex, laparoscopy [7,10], laparotomy, cloacal examination, determination of steroids from faeces, eggs [7], or cytogenetic analyses [1,11]. Disadvantages of these methods are running time, costs, and in some cases, the fact that they are invasive and damaging [7,8,9,10].

### 1.1. Molecular Methods for Sexing Birds

Molecular methods are designed to eliminate the drawbacks of the classical methods mentioned earlier. The *CHD1* gene-encoding DNA chromo-helicase binding protein 1 was the first gene discovered on the avian W chromosome (CHD1-W). CHD1-Z is located on the Z chromosome in both sexes, thus being a gametologous gene. *CHD1-Z* and *CHD1-W* genes are sexually dimorphic genes conserved to the majority of independently evolving avian species from Neognathae clade (non-ratites) but not in Palaeognathae (ratites and tinamous). The protein structures of CHD1-Z and CHD1-W are very similar to each other in terms of amino acid sequences. The helicase domain of the *CHD1* gene is highly conserved. In Palaeognathae birds these gametologous genes cannot be distinguished between them. Avian gametologs share common descent from homologous autosome pairs. There is no recombination between the two genes, and also no autosomal copies were detected [12]. Accordingly, specific protocols have been developed to provide a DNA test for sex determination for these birds [13,14].

Molecular sexing involves PCR amplification of Z and W alleles using specific primers that are designed to detect intron variations in them. The most frequently used primers are P2/P8, CHD1F/CHD1R, 1237L/1272H, and 2550F/2718R, which are related to the *CHD1* gene [4,7,15,16,17,18]. Amplified products would need to migrate as a single band in the male sample, males (ZZ), because the copies of the gene from two Z chromosomes are identical in length, while in females (ZW), two bands appear because Z and W copies of the gene have length polymorphism [19,20,21].

Various specific techniques used alongside PCR, namely SSCP [11], RFLP [17], AFLP [22], microsatellites [11], AS-PCR [23,24], capillary electrophoresis [15,25], qPCR combined with melting curve analysis [13,20,26,27], or HRM analysis [28,29,30], have been developed to ensure the applicability of molecular sex diagnosis techniques to as many bird species as possible. The main advantages and disadvantages of these techniques are listed in Table 1.

Difficulties in *CHD1*-based molecular sexing caused by intraspecific variations at the *CHD1-Z* homologs detected in the amplified intronic region have been reported when primers CHD1F/CHD1R, P2/P8, and 2550F/2718R were used, with better results for CHD1F/CHD1R, P2/P8 [37].

Nevertheless, with advancements in real-time PCR and genome-wide analysis methods, the detection and analysis of novel specific markers and protocols have been made easier [2,19].

### 1.2. Genes That Are Linked to Bird Sexing

In addition to the *CHD1* gene, there are more than 40 gametologous genes, and some of them have been studied for sex differentiation in birds [11]. Other examples are *CHRNA6*, *DDX4*, *VPS13A*, *LPAR1*, *TMEM161B*, and Z-specific genes that were successfully amplified in 17 Neognathae bird species, with better results for *TMEM161B* and *DDX4* (14/17 species) [36]. In a large study across 70 Neognathae species and three Palaeognathae species, it was concluded that *CHRNA6*, *DDX4*, *TMEM161B*, and *VPS13A* genes can reveal sex in Neognathae birds. In the Palaeognathae birds, the *DOCK8*, *FUT10*, *PIGG*, and *PSD3* genes can be used to identify sex, while the *LPAR1* gene has a higher accuracy in identifying sex in both clades [38]. In consequence, a logistic regression model based on quantitative qPCR values is proposed as an innovative approach [39].

The molecular mechanism of sex determination in birds is complex, being governed by genetic and hormonal interactions. Two theories have been postulated to explain how genes located on the sex chromosomes regulate gonadal differentiation and subsequent sexual development. One theory posits that the trigger factor for the differentiation of pregonads into gonads is the dosage of genes located on the Z chromosome (e.g., *DMRT1* gene). In contrast, an alternative theory asserts that W-linked genes serve as determinants of differentiation into ovarian tissue and inhibit the development of testicular tissue [40]. Some genes (e.g., *CYP19A1, FOXL2, WNT4*) have been observed to exhibit higher levels of expression in female gonads, while other genes (e.g., *DMRT1, SOX9, AMH*) demonstrate notably higher levels of expression in male gonads [41]. The *DMRT1* gene, located on the Z chromosome, plays a pivotal role in avian sex determination. A single functional copy of the *DMRT1* gene has been demonstrated to induce the development of ovaries in ZZ chickens, as reported in a study utilizing the CRISPR-Cas9 technique [42]. Sex hormones have been demonstrated to play a significant role in the process of sexual differentiation. The role of these subjects is most clearly elucidated in cases of hormonally induced sex reversal [41].

New potential sex-linked markers, such as retroposon, mobile elements that propagate and integrate randomly in genomes via RNA intermediates, were studied [29]. The gametologous retroposon insertions which give distinct size differences in Z and W amplicons (e.g., 200 bp in CHD1 intron 16, 400 bp in NIPBL intron 16, and 500 bp in CHD1 intron 9) can be used as tools for examination of the relative chronology of gametologous genes and for differentiation of molecular gender in Neognathae birds.

Few studies have examined whether primers developed for a particular species could be efficiently used across a broad range of taxonomically different bird families. One particularly promising primer set is the one designed for the domestic pigeon (*Columba livia*), the pCHD1F/pCHD1R primers [2]. Liang et al. demonstrated 100% success with these primers in pigeons, but their utility in other bird groups had not been fully assessed [2].

The reason for this molecular study was a practical need, specifically for pet bird owners to identify the gender of their birds with certainty. Also, the western region of Romania has many pigeon breeders, and we included pigeons in this study to test the molecular sex of this species using both pigeon-specific primers and universal primers. The aim of this study was to validate a simple, rapid, low-cost classic PCR method that can be used for molecular sex identification in the main bird species of veterinary professional interest.

## 2. Results

**Figure 1:**
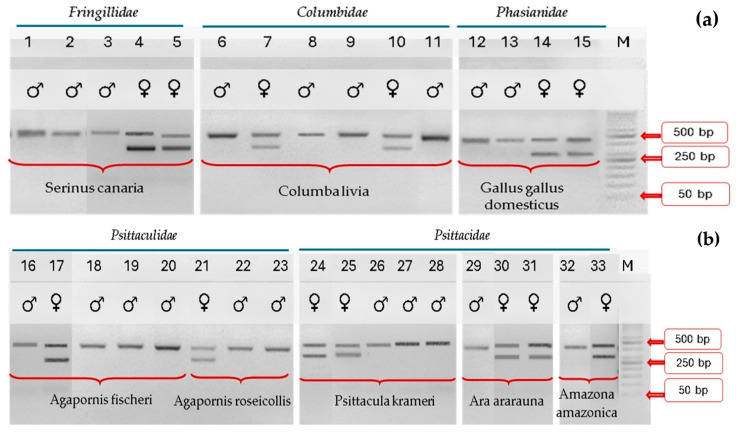
Electrophoretic migration (**a**) of *Fringillidae*, *Columbidae*, and *Phasianidae* samples and (**b**) *Psittaculidae* and *Psittacidae* samples after using universal primer sets CHD1F/CHD1R. The CHD1F/CHD1R- amplified products demonstrate the presence of one band for males and two bands for females. M = DNA size ladder (VWR, K180-500, ready-to-use; VWR International SAS, Paris, France). Cropped gels are shown.

**Figure 2:**
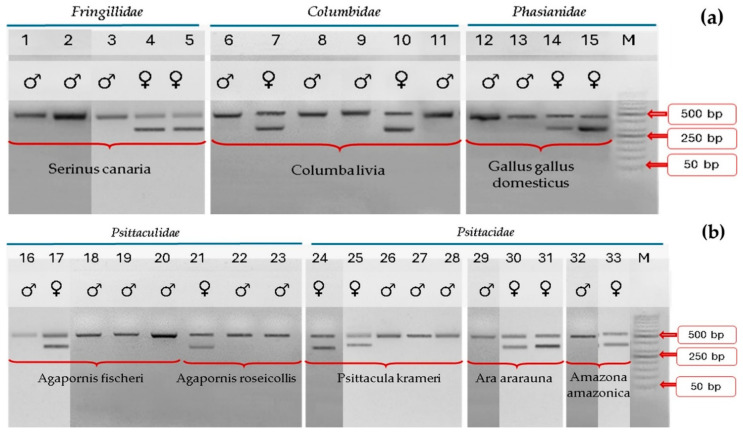
Electrophoretic migration (**a**) of *Fringillidae*, *Columbidae*, and *Phasianidae* samples and (**b**) *Psittaculidae* and *Psittacidae* samples after using pigeons primer sets pCHD1F/CHD1R. The pCHD1F/CHD1R-amplified products demonstrate the presence of one band for males and two bands for females. M = DNA size ladder (VWR, K180-500, ready-to-use; VWR International SAS, France). Cropped gels are shown.

Of the genotyped birds 39.39% were females, and 60.61% were males. The results of the analysis of the *CHD1* gene fragment amplified using both the universal and pigeon primer sets CHD1F/CHD1R for all sampled individuals indicated on the agarose gel a single band in males (CHD1-Z) and two bands in females (CHD1-Z and CHD1-W) (Figure 1 and Figure 2).

The length of the *CHD1-Z* and *CHD1-W* gene fragments amplified with the two different sets of primers is shown separately for each species (Table 2).

## 3. Discussion

Our results demonstrate that the pigeon-derived CHD1 primer pair (pCHD1F/pCHD1R) efficiently amplified sex-linked *CHD1* gene fragments in bird species from five distinct avian families. The consistent detection of sex-specific amplicons in all tested species suggests that the primer binding sites within the *CHD1* gene are well-conserved across phylogenetically distant taxa. This observation reinforces previous findings that highlight the stability of the CHD1 intronic regions targeted by molecular sexing primers. For instance, Kroczak et al. [18] showed that the CHD1i9 marker can be successfully used for sex identification even in Palaeognathae birds, indicating the broader potential of CHD1-based markers beyond their original target species.

In this context, the present study offers further evidence that primers initially designed for *Columba livia* can be applied reliably across a taxonomically diverse set of birds without requiring species-specific redesign. Under uniform PCR conditions and using a standardized, non-invasive sampling method, the pCHD1F/pCHD1R primer pair yielded clear and reproducible banding patterns in representatives of *Psittaculidae*, *Psittacidae*, *Columbidae*, *Fringillidae*, and *Phasianidae*. The amplification results were supported by in silico simulations of primer binding and multiple sequence alignments, which confirmed the high degree of conservation in the targeted CHD1 regions. These findings underscore the practical advantages of using a single, robust primer set for molecular sexing across multiple bird species, streamlining laboratory workflows and reducing both time and cost in diagnostic or breeding-related applications.

The conserved sequence within the *CHD1* gene across species is the main factor that explains why CHD1 primers designed from *Columba livia* are compatible with other non-ratite birds [43]. Nonetheless, the accuracy of primer binding could be impacted by the sequencing differences between various avian breeds.

Our findings align with previous research that has established PCR-based sex determination as a widely accepted and effective technique for birds, particularly through the amplification of the *CHD1* gene located on the Z and W chromosomes [19,43,44]. The CHD1F/CHD1R primer set is widely recognized as a universal marker for molecular sexing, as demonstrated by Çakmak et al. [37]. A key methodological difference between our study and that of Çakmak et al. was the electrophoresis gel concentration [37]. While Çakmak et al. utilized a 3% agarose gel, we performed our analysis using a 1.8% agarose gel, which allows better migration of larger DNA fragments. This could explain why our study yielded slightly different fragment size ranges (440–536 bp). Additionally, species-specific intronic length polymorphisms within the *CHD1* gene contribute to natural variation in fragment sizes among birds [34]. Given that the *CHD1* gene contains non-coding regions that vary in length across species, even studies using the same primer sets may report different fragment sizes.

In our study, the performance of the pigeon-specific pCHD1F/pCHD1R primers was comparable to that of widely used universal primer sets such as P2/P8, with clear high-resolution bands. This equivalence is likely explained by the high degree of conservation within the intronic regions of the *CHD1* gene across the Neognathae species included in the present work. Therefore, while P2/P8 remain strong universal markers, our results indicate that primers originally developed for *Columba livia* can be reliably applied across multiple avian families without additional optimization. This reinforces the flexibility of CHD1-based molecular sexing and highlights the conserved nature of the CHD1 target region.

DNA concentration plays a crucial role in determining PCR efficiency, particularly when working with buccal swab samples, which may yield limited amounts of genomic material. In our study, we observed that when the total DNA quantity used per reaction was below 20 ng, amplification was often unsuccessful, resulting in absent or faint bands on the agarose gel. In contrast, when DNA input exceeded 20 ng per reaction, clear and reproducible amplification was consistently obtained. These findings suggest that, under our experimental conditions, a minimum of 20 ng of template DNA per PCR reaction is required to ensure reliable results. This threshold should be taken into account when using non-invasive sampling techniques and emphasizes the importance of optimizing DNA extraction protocols to obtain sufficient yields.

Some studies have reported species-specific anomalies, where both sexes display a single band of different sizes, making sex differentiation challenging [45]. Çakmak et al. observed such anomalies in *Meleagris gallopavo* (turkey), *Coturnix coturnix* (quail), *Psittacula alexandri*, and 10 species of Passeriformes [37]. In contrast, our study did not encounter such anomalies; all birds from the five different families analyzed exhibited clear, sex-specific banding patterns, allowing accurate molecular differentiation between males and females. The oral swabs method of sampling seems to have advantages, such as short time handling the bird, no dangerous tools, and ease of processing in the lab, compared with plucked feathers [46,47].

In *Psittaculidae* species (*A. fischeri* and *A.roseicollis*), both CHD1F/CHD1R and pigeon CHD1F/CHD1R primers successfully amplified CHD-Z and CHD-W fragments, yielding distinct banding patterns between males and females.

Similar results for *Agapornis birds*, but by using different primers (P2, NP and MP), one clear DNA band in size of 400 bp for the Z chromosome (ZZ) and one band of 350 bp for the W chromosome along with one band of 400 bp for the Z chromosome, were observed (ZW) [48]. In the masked lovebird (*Agapornis personata*), the same clear bands were observed in male and female lovebirds through the classic PCR method [49].

In the *Psittaciformes* species, the universal sexing marker is hard to have because of the different patterns of PCR products, depending on the tested species, as stated in the study of Kroczak et al., suggesting that the use of multiple markers (CHD1i16, NIPBLi16, CHD1i9, and CHD1iE) decreases the chance of misidentification of sex [21]. CHD1-Z specific fragments were visible, and the differences between the length of fragments amplified with universal primers and fragments amplified with pigeon primers were minor. For *S. canaria*, PCR amplification using CHD1F/CHD1R and pigeon CHD1F/CHD1R generated clear, species-specific fragment sizes. Canaries, like many other species from the *Fringillidae* family, show high success rates with CHD1-based sexing, as they exhibit sufficient sequence homology in the *CHD1* gene, which has been previously confirmed by Griffiths et al. [43]. Successful sexing of species of the order Psittaciformes was obtained with primers P8/P2 and with primers 2550F/2718R [50]. Our findings for *C. livia* (*Columbidae* family) are consistent with Liang et al., who reported CHD1-Z and CHD1-W fragment sizes of 474 bp on the Z chromosome and 319 bp on the W chromosome [2].

For *G. gallus*, our study results align with the published literature [30]. Although chickens are more distantly related to pigeons, CHD1 primers developed for pigeons can be applied to chickens with success. Given that chickens are commonly used as a reference species in molecular sexing, our findings further validate the consistency and accuracy of these primer sets.

Depending on the bird species, the size of the alleles varies; for the Z allele it ranges from 246 to 396 bp, while for the W allele a variation of 254 to 412 bp is expected. The expected and specific sizes of the bands in parrots (*Amazona aestiva*), representative of the alleles, are allele Z–396 bp and allele W–412 bp [2,37].

While CHD1F/CHD1R and pCHD1F/CHD1R were 100% efficient in our study, certain limitations should be considered. The initial constraint of the study is the limited number of samples/bird families. However, we are optimistic that, given the interest of pet bird owners in sexing, the number of samples will increase in the future, thereby ensuring a more robust validation of the results. Agarose gel electrophoresis, though widely used, has limited resolution when fragment sizes differ by only a few base pairs. Minimizing the errors of an experiment is very important to have results with high accuracy. Factors such as the concentration of buffer solution during electrophoresis or the concentration of agarose can influence the success of a protocol [3]. In qPCR amplification, working conditions are also very important, such as the quantity and quality of Taq polymerase enzymes [3] or DNA quality [36].

Like any technique, the method of sexing birds using PCR must be modified for certain species. This is especially important for bird species that have a small difference between the CHD1-Z and CHD1-W amplicons. The limitation of our study is the small number of samples. Future studies with a larger number of samples for each species and sequencing of the amplified fragments will help to complement the encouraging results obtained from this study.

Our results demonstrated that CHD1F/CHD1R and pCHD1F/CHD1R primer sets successfully amplified fragments of the *CHD1* gene in all 33 analysed samples, confirming its reliability in PCR-based sex identification methods in birds. The general applicability of pigeon CHD1 primers underscores their value in avian genetic research. The sex diagnosis of all examined samples from five bird families of different ages was confirmed with certainty by the molecular method.

## 4. Materials and Methods

All sampling protocols were followed in accordance with the relevant national guidelines and regulations. The collection of the biological samples was performed with the consent of the pets’ owners, according to the code of the Romanian Veterinary College (protocol numbers 34/01.12.2012) and the procedures of the University Veterinary Clinics of the Faculty of Veterinary Medicine, Timisoara.

**Figure 3:**
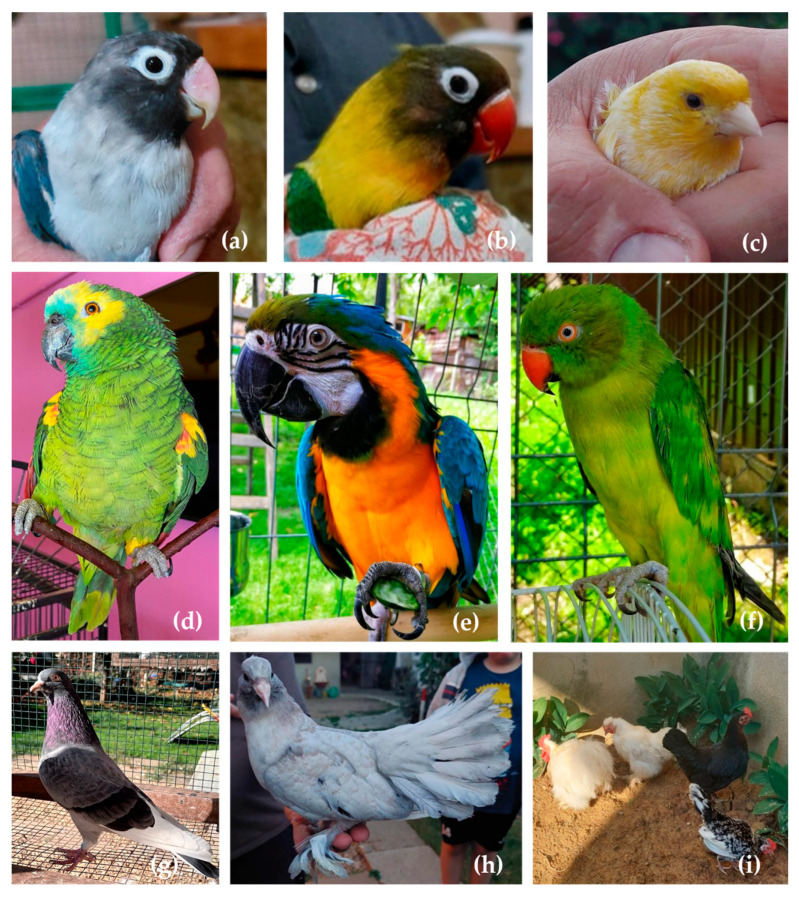
Illustrative photographs of pet bird species used in the study: (**a**) Fisher’s lovebird (*Agapornis fischeri*), (**b**) lovebird (*Agapornis roseicollis*), (**c**) canary (*Serinus canaria*), (**d**) orange-winged amazon (*Amazona amazonica*), (**e**) blue and yellow macaw (*Ara ararauna*), (**f**) rose-ringed parakeet (*Psittacula krameri*), (**g**) English Tippler pigeon (*Columba livia*), (**h**) Indian fantail pigeon (*Columba livia*), and (**i**) hen (*Gallus gallus domesticus*), original pictures.

The biological material used for the study came from 5 different bird families (the *Psittaculidae* family, the *Psittacidae* family, the *Columbidae* family, the *Fringillidae* family, and the *Phasianidae* family) (Figure 3).

From the *Psittaculidae* family, *Agapornis* genus, there were five samples of *Agapornis fischeri* (Fisher parrot), and three samples from *Agapornis roseicollis* (lovebird), while from the *Psittacula* genus, five samples from *Psittacula krameri* (rose-ringed parakeet). From the *Psittacidae* family, *Ara* genus, there were three samples of *Ara ararauna*, and two samples from *Amazona amazonica.* From the *Columbidae* family, *Columba* genus, there were six samples of *Columba livia* (three English Tippler and three Indian Tippler). From the *Fringillidae* family, *Serinus* genus, there were five samples of *Serinus canaria,* while from the *Phasianidae* family, *Gallus* genus, there were four samples of *Gallus gallus domesticus.*

The samples were handled with surgical gloves, collected using sterile pharyngeal exudate collectors. The procedure consisted of inserting the collector into the bird’s oral cavity and then performing several vigorous rotating movements to obtain a high number of cells. Out of the total samples, 4 samples (ID 25, 26, 30 and 31) were contour feathers with intact calamus. The collectors with the biological samples were labelled and stored at −20 °C until processing.

### DNA Isolation, PCR Reaction, and Electrophoresis

DNA extraction was performed from buccal swabs using the NucleoSpin DNA Forensic Kit (Macherey-Nagel, Düren, Germany), following the manufacturer’s instructions.

The DNA obtained was evaluated qualitatively and quantitatively by the spectrophotometric method using Nano Drop 8000 spectctrophotometer (Thermo Scientific, Waltham, MA, USA). The absorbance ratios of 260/280 nm and 260/230 nm were used as indicators of DNA purity. Samples showing a 260/280 ratio between 1.8 and 2.0 and a 260/230 ratio of ≥1.8 were considered acceptable for downstream PCR analyses, which reflects low protein and organic compound contamination (Supplementary Table S1).

Oligonucleotides used as PCR primer sets CHD1F/CHD1R were TATCGTCAGTTTCCTTTTCAGGT and CCTTTTATTGATCCATCAAGCCT, as previously reported by Cakmak et al. [37], and primer sets CHD1F/CHD1R for pigeons were TTCTGAGGATGGAAATGAGT and GCAATGGTTACAACACTTC, as previously reported by Liang et al. [2].

PCR reactions were carried out using an Eppendorf Mastercycler pro S Thermal Cycler (Eppendorf, Hamburg, Germany). Each 25 µL reaction mixture contained 12.5 µL GoTaq^®^ Green Master Mix (Promega, Madison, WI, USA), 10 µM of each primer, and >20 ng of genomic DNA per reaction, with PCR-grade water added to a final volume of 25 µL. For the amplification of CHD1F/CHD1R universal primers, we followed a touchdown PCR protocol, in which the annealing temperature was decreased by 1 °C per cycle, starting from 57 °C until reaching 50 °C, followed by 30 additional cycles at this final annealing temperature. The reaction concluded with a final extension at 74 °C for 5 min, as proposed by Çakmak et al. (2016) [37]. For the pCHD1F/pCHD1R primer set, the PCR programme was carried out under the following conditions: an initial denaturation at 94 °C for 5 min, followed by 38 cycles consisting of denaturation at 94 °C for 30 s, annealing at 53.5 °C for 30 s, and extension at 72 °C for 30 s, as proposed by Liang et al. [2].

For all PCR runs, a no-template negative control was included to monitor potential contamination. These controls did not produce any detectable amplification. As the focus of the study was on the amplification pattern of the CHD1 fragments in avian samples, negative control lanes are not shown in the electrophoresis images.

Prior to analysis, *CHD1* gene sequences for five avian species were retrieved from the NCBI Nucleotide database, and in silico PCR amplification was simulated using the SMS2 PCR Products tool from the Bioinformatics.org suite [51] to validate binding sites of the pigeon primers across species. The reference CHD1Z sequences used for the comparative analysis were as follows: *Columbidae*–KM593258.1, *Psittaculidae*–NC_047557.1, *Psittacidae*–NW_026901617.1, *Phasianidae*–AC186875.2, and *Fringillidae*–NC_066343.1. These reference sequences were subsequently aligned to confirm primer binding site conservation among the five avian families examined in this study.

PCR products were separated by electrophoresis on a 1.8% agarose gel prepared in standard TAE buffer and run at 80–100 V for approximately 1 h. The DNA fragments were stained with ethidium bromide and visualized under UV illumination using a gel documentation system (UVP, England). A 100 bp molecular weight marker was used to estimate the size of the PCR amplicons. Band positions were analysed using GelAnalyzer 23.1.1 software [52], allowing for improved accuracy in determining fragment sizes.

The fragment size data presented in Table 2 reflect approximate lengths obtained through two complementary approaches. First, gel-based estimations were performed as described above, using the migration patterns of the PCR products in relation to the DNA ladder. Second, an in silico analysis was carried out using publicly available *CHD1* gene sequences retrieved from the NCBI Nucleotide database in order to simulate the expected amplification regions for the pigeon-specific primers (pCHD1F/pCHD1R). These simulations were conducted with the SMS2 PCR Products tool from the Bioinformatics.org suite [51].

## 5. Conclusions

This study provides the first systematic evaluation of pigeon-specific CHD1 primers (pCHD1F/pCHD1R) for molecular sexing in five avian families of veterinary relevance: *Psittaculidae*, *Psittacidae*, *Columbidae*, *Fringillidae*, and *Phasianidae*. The consistent amplification of sex-specific CHD1 fragments using a single primer set highlights the cross-species compatibility of these primers, reducing the need for species-specific designs.

The methodology, based on non-invasive sampling and classic PCR, proves to be an accessible and cost-effective solution suitable for both clinical and breeding applications. Multiple sequence alignments confirmed the conservation of primer binding regions across taxonomic groups, while the use of universal CHD1F/CHD1R primers served as an internal reference to validate fragment size ranges. The ability to obtain clear results regardless of age or taxonomic family underscores the utility of this approach in veterinary practice. Fragment size differences observed across species were attributed to intronic variability and gel resolution. Moreover, we found that a DNA concentration threshold of 20 ng/µL is necessary for reliable amplification, emphasizing the importance of maintaining adequate sample quality when using a non-invasive method. Overall, this protocol supports accurate, reproducible molecular sexing, offering a practical tool for routine application in avian veterinary diagnostics.

## Figures and Tables

**Table 1:** The PCR-based methods used in avian molecular sexing.

Methods	Species in Which It Was Applied	Advantages	Disadvantages	References
SSCP	goshawk (*Accipiter gentilis*), cooper’s hawks (*Accipiter cooperii*)	enhance the resolution enhance sensibility in the detection of small variations between Z and W alleles	time-consuming (requires optimization of several factors) moderately laborious	[30,31,32]
RFLP	brown skua (*Catharacta lonnbergi*), Sfinx’s makaw (*Cyanopsitta spixii*)	simple method	time-consuming (requires selection of an appropriate restriction enzyme)	[1,7,22,30]
RAPD	Chinese geese (*Anser cygnoides*), white Roman geese (*Coscoroba coscoroba*), and Landaise geese	simple method	reduced reproducibility and sensitivity relatively expensive (requires species-specific nature of markers to increase reproducibility and sensitivity)	[30,33]
AFLP	ostrich (*Struthio camelus*), cormorant (*Phalacrocorax aristotelis*), shag (*Phalacrocorax aristotelis*)	10 times more powerful than RAPD, producing 50–100 bands stability of the reaction increase power	high processing time relatively expensive (requires species-specific nature of markers)	[22,30]
Microsatellites/STRs/SSRs	American cranes (*Grus americana*), falcons	easily detected	requires species-specific markers, which increase the development time and intensity of labour	[30]
AS-PCR/ARMS	black swans (*Cygnus atratus*); black kite (*Milvus migrans*), Northern goshawk (*Accipiter gentilis*), Eastern marsh harrier (*Circus spilonotus*), peregrine falcon (*Falco peregrinus*) etc	simple method useful and rapid method similar with standard PCR-based protocols	-	[23,24,30]
Capillary electrophoresis	fairy pitta (*Pitta nympha*), Jungle fowl (*Gallus gallus*), mute swan (*Cygnus olor*) etc.	simple high speed high-throughput applicability high resolution and sensitivity automated workflow and data acquisition	limited intraspecies variation and reproducibility	[30,34]
qPCR	domestic pigeon (*Columba livia domestica*), budgerigar (*Melopsittacus undulatus*), lovebird (*Agapornis roseicollis*), rose-ringed parakeet (*Psittacula krameri*), African grey parrot (*Psittacus erithacus*), red-rumped parrot (*Psephotus haematonotus*), etc.	high sensitivity/specificity efficiency and rapid execution	relatively expensive (the necessity of species-specific probes)	[20,30,35,36]
Real-time PCR combined with melting curve analysis	Eurasian pygmy owls (*Glaucidium passerinum*), Japanese quail (*Coturnix japonica),* eastern screech-owls *(Megascops asio*), etc.	rapid execution sensitivity and high-throughput applicability	time-consuming (careful selection of the primer sets according to the species analysed)	[4,13,28,30]
HRM	common quail (*Coturnix coturnix*), Japanese quail (*Coturnix japonica*)	high sensitivity high specificity excellent thermal stability low cost relative to the traditional methods	specific HRM software	[28,29,30]

SSCP = single-strand conformation polymorphism; RFLP = restriction fragment length polymorphism; RAPD = random amplified polymorphic DNA; AFLP = amplified fragment length polymorphism; STR = short tandem repeats; SSR = simple sequence repeats; AS-PCR = allele-specific PCR; ARMS = amplification refractory mutation system; qPCR = real-time quantitative PCR; HRM = high-resolution melting.

**Table 2:** Size of amplified *CHD1* gene fragments obtained with primer sets CHD1F/CHD1R and pigeon CHD1F/CHD1R, differentiated by sex and avian family.

Family	Species	Sex	CHD1F/CHD1R (bp)		pCHD1F/p.CHD1R (bp)	
			Z	W	Z	W
*Fringillidae*	*Serinus canaria*	M	508–529	-	452–533	-
		F	502–520	311	465–471	312–316
*Columbidae*	*Columba livia*	M	477–515	-	443–469	
		F	496	297–309	460–469	311–323
*Phasianidae*	*Gallus gallus*	M	440–458	-	469–486	-
		F	458	308	486	324
*Psittaculidae*	*Agapornis fischeri*	M	506–536	-	477–504	-
		F	515	331	476	315
	*Agapornis roseicollis*	M	515–525	-	452–469	-
		F	515	324	469	329
	*Psittacula krameri*	M	514–519	-	470	-
		F	515	328–334	469	306–327
*Psittacidae*	*Ara* *ararauna*	M	496	-	460	-
		F	508–510	338	458	324–329
	*Amazona* *amazonica*	M	514	-	418	-
		F	528	339	504	330

p = pigeon; Z = fragment size corresponding to *CHD1-Z* gene; W = fragment size from *CHD1-W* gene; F = female sample; M = male sample

## Data Availability

The original contributions presented in this study are included in the article/Supplementary Material. Further inquiries can be directed to the corresponding authors.

## Supplementary Materials

The following supporting information can be downloaded at: https://www.mdpi.com/article/10.3390/ijms262211142/s1.
