# Supplementary material for: Cross-Species Validation of Pigeon-Specific CHD1 Primers for Molecular Sexing in Pet Birds
Source: Int J Mol Sci. 2025 Nov 18;26(22):11142. doi: 10.3390/ijms262211142 (PMC12652837; doi:10.3390/ijms262211142)
Supplement: Supplementary file 1 [file ijms-26-11142-s001.zip › ijms-3983550-supplementary.pdf]

Cross-species validation of pigeon specific CHD1 primers for molecular sexing in pet birds

Simona Marc[1], Oana Maria Boldura[1*], Cristina Paul[2*], Maria Roberta Tripon[3], Gabriel Otavă[1] and Jelena Savici[1]

[1] Faculty of Veterinary Medicine, University of Life Sciences "King Mihai I" from Timisoara, Calea Aradului 119, 300645 Timisoara, Romania; simona.marc@usvt.ro (S.M.); jelenasavici@usvt.ro (J.S.); oanaboldura@usvt.ro (O.M.B.); gabrielotava@usvt.ro (G.O.)

[2] Faculty of Chemical Engineering, Biotechnologies and Environmental Protection, Politehnica University Timisoara, Vasile Pârvan No. 6, 300223 Timisoara, Romania; cristina.paul@upt.ro (C.P.)

[3] Faculty of Engineering and Applied Technologies, University of Life Sciences "King Mihai I" from Timișoara, Calea Aradului No. 119, 300645 Timișoara, Romania; roberta.tripon@usvt.ro (M.R.T.)

* Correspondence: oanaboldura@usvt.ro, cristina.paul@upt.ro; Tel.: +40-256-404234

Table S1. Qualitative and quantitative values for DNA suspensions

Sample no.	DNA quality ratio (260/280)	DNA Quantity ng/ μL (spectrophotometric method)
1	1.88	2.07
2	1.78	32.07
3	2.01	136.1
4	1.93	39.8
5	1.85	36.56
6	1.87	14.63
7	1.72	11.64
8	1.75	15.82
9	1.66	5.07
10	1.70	9.97
11	1.62	5.27
12	1.76	77.13
13	1.62	65.14
14	1.76	9.60
15	1.91	8.21
16	1.59	5.02
17	1.76	47.75
18	1.62	30.83
19	1.81	12.52
20	1.78	30.24
21	1.75	23.00
22	1.68	3.89
23	1.71	15.93
24	1.93	5.00
25	1.95	6.15
26	1.65	5.89
27	1.87	19.47
28	1.72	48.33
29	1.95	6.30
30	1.60	3.598
31	1.58	4.16
32	1.65	20.69
33	1.59	7.23
